# Feature Genes Selection Using Fuzzy Rough Uncertainty Metric for Tumor Diagnosis

**DOI:** 10.1155/2019/6705648

**Published:** 2019-01-27

**Authors:** Jiucheng Xu, Yun Wang, Keqiang Xu, Tianli Zhang

**Affiliations:** ^1^College of Computer and Information Engineering, Henan Normal University, Xinxiang, Henan, China; ^2^Engineering Technology Research Center for Computing Intelligence and Data Mining, Xinxiang, Henan, China; ^3^College of Political Science and Public Administration, Henan Normal University, Xinxiang, Henan, China

## Abstract

To select more effective feature genes, many existing algorithms focus on the selection and study of evaluation methods for feature genes, ignoring the accurate mapping of original information in data processing. Therefore, for solving this problem, a new model is proposed in this paper: rough uncertainty metric model. First, the fuzzy neighborhood granule of the sample is constructed by combining the fuzzy similarity relation with the neighborhood radius in the rough set, and the rough decision is defined by using the fuzzy similarity relation and the decision equivalence class. Then, the fuzzy neighborhood granule and the rough decision are introduced into the conditional entropy, and the rough uncertainty metric model is proposed; in the meantime, the definition of measuring the significance of feature genes and the proof of some related theorems are given. To make this model tolerate noises in data, this paper introduces a variable precision model and discusses the selection of parameters. Finally, based on the rough uncertainty metric model, we design a feature genes selection algorithm and compare it with some existing similar algorithms. The experimental results show that the proposed algorithm can select the smaller feature genes subset with higher classification accuracy and verify that the model proposed in this paper is more effective.

## 1. Introduction

Nowadays, with the continuous changes of human lifestyle and environment, the incidence and mortality of cancers are rising. Therefore, how to improve the analysis, identification, and treatment of tumors has become one of the research hotspots of scholars [1]. Gene expression profiling data can approximately reflect the expression information of the entire genome of biological cells. With the development of gene chip technology, the accurate acquisition of gene expression profiling data becomes possible, which provides an important basis for clinical tumor diagnosis and tumor pathogenesis research [2–4]. However, among the large number of genes included in the gene expression profiling data, there are only a few important genes that can be used as information genes to track diseases [5, 6]. Therefore, when scholars process gene expression profiling data, they will be transformed into information genes selection problems, that is, feature genes selection, whose purpose is to reduce noises and redundant data in gene expression profiles and to obtain feature genes subsets with strong disease-recognition ability [5].

The gene expression profiling data contain complex and specific information. If the original information of the data can be applied to the calculation as accurately as possible, the result of feature genes selection will be improved to a large extent. The classical rough set proposed by Pawlak [7] has been extensively developed and studied [8, 9]. Its theory is based on the equivalence relation and can only deal with the discrete data. The processing of continuous data needs to be discretized, and it will face problems just like information loss. To solve this problem, neighborhood rough set [10–13] and fuzzy rough set [14–18] are successively proposed as two important models. The neighborhood rough set can directly process the continuous data, which overcomes the shortcomings of classical rough set, but it cannot accurately describe the fuzziness of samples under the fuzzy background. In the fuzzy rough set, the description of a sample is usually depicted by its relationship with the neighbor samples, so the data noise will increase the risk of the calculation result and increase the classification error rate [19]. For this problem, the concept of fuzzy neighborhood is proposed in the literature [20], which overcomes the above deficiencies to some extent and constructs a fitting feature selection model based on the fuzzy rough set. It is crucial to find superior feature evaluation functions in the feature selection process, such as the dependence [19, 21, 22] and information entropy [23–25] methods that have already been proposed. As a kind of knowledge acquisition tool, rough set theory is gradually used in the analysis of gene expression profiling data, which uses the dependency function to evaluate the classification ability of feature genes subset, and has achieved good research results. However, the dependence function mainly depends on the positive domain and the boundary domain, which will lead to inaccurate measurement. At present, some scholars have introduced information entropy into rough sets, such as rough entropy [26] and conditional entropy [27, 28]. What some scholars mentioned in the literatures [29, 30] is that the algebraic definition of attribute importance focuses on the influence of attributes on the certain subset of categories, while the information theory definition considers the influence on the uncertain subset of categories, which are highly complementary. Therefore, the combination of the two will make the measurement mechanism more comprehensive.

In order to make the measurement mechanism more comprehensive and reduce the loss of original data information in the calculation process, this paper uses the combination of fuzzy and neighborhood concepts in the data characterization stage to redefine the fuzzy neighborhood granule in the literature [19] and uses the fuzzy similarity between samples and the decision equivalence class to define the rough decision. Based on the above concept, the original information of the data can be restored as perfectly as possible during the sample characterization. Then, conditional entropy is introduced when the feature evaluation function needs to be selected. A new feature genes selection model is proposed: the rough uncertainty metric model (RUM). At the same time, the definition of the evaluation function for the significance of feature genes and the proofs of some related theorems are given. Finally, based on the rough uncertainty metric model, a feature genes selection algorithm is designed and compared with other existing similar algorithms to prove the validity of the new model.

The remainder of the paper is organized as follows. In Section 2, some basic concepts about rough set theory are reviewed. In Section 3, the rough uncertainty metric model is proposed and a heuristic feature genes selection algorithm is presented for this model. In Section 4, the validity and feasibility of the proposed model are verified by comparing experiments.  Section 5 concludes the paper.

## 2. Related Theoretical Knowledge

This section mainly reviews some basic conceptual knowledge of neighborhood rough set theory and fuzzy rough set theory, including neighborhood relation, fuzzy relation, and the combination of fuzzy and neighborhood concepts.

### 2.1. Neighborhood Relation

In the data processing stage, the neighborhood rough set mainly uses the neighborhood radius to realize the division of the universe. It can control the size of the sample neighborhood and process the continuous numerical data through the relationship measurement between samples. The basic neighborhood concepts proposed by Hu et al. [10] is as follows:

Let the universe of discourse *U*={*x*_1_, *x*_2_,…, *x*_*n*_} be an *n*-dimensional real-value space, *L*=*R* ^*n*^ × *R* ^*n*^⟶*R*. Then, *L* is a measure on *R* ^*n*^ and satisfies the following conditions:

1. *L*(*x*_1_, *x*_2_) ≥ 0, and the equality holds up if and only if *x*_1_=*x*_2_, ∀*x*_1_, *x*_2_ ∈ *R* ^*n*^
2. *L*(*x*_1_, *x*_2_)=*L*(*x*_2_, *x*_1_), ∀*x*_1_, *x*_2_ ∈ *R* ^*n*^
3. *L*(*x*_1_, *x*_3_) ≤ *L*(*x*_1_, *x*_2_)+*L*(*x*_2_, *x*_3_), ∀*x*_1_, *x*_2_, *x*_3_ ∈ *R* ^*n*^

For ∀*x*_*i*_⊆*U*, its neighborhood is expressed as *ε*(*x*_*i*_)={*x* | *x* ∈ *U*, *L*(*x*, *x*_*i*_) ≤ *ε*} and *ε* > 0. According to the nature of metric,

1. *ε*(*x*_*i*_) ≠ ∅
2. *x*_*j*_ ∈ *ε*(*x*_*i*_)⟹*x*_*i*_ ∈ *ε*(*x*_*j*_), ∀*x*_*j*_⊆*U*
3. ∪_*i*=1_^*n*^ *ε*(*x*_*i*_)=*U*

It can be seen from (3) that *U* is the full coverage of {*ε*(*x*_*i*_)|, *i*=1,2,…, *n*}.

### 2.2. Fuzzy Relation

The fuzzy relation is described approximately by the membership degree of a sample on a set about an attribute. This expression is a good mathematical representation of clear and unclear concepts. Dubois and Prade [14] mentioned the basic fuzzy relations.

Let *U*={*x*_1_, *x*_2_,…, *x*_*n*_} and a mapping be *S*(·) : *U*⟶[0,1] on the universe of discourse *U*, then *S* is a fuzzy set. For ∀*x* ∈ *U*, *S*(*x*) is the membership of *x* on *S*, and *F*(*U*) is recorded as a fuzzy set on *U*. *B* is an attribute set of the sample, which can induce a fuzzy binary relationship *R*_*B*_ on *U*.

If ∀*M*, *N*⊆*F*(*U*) are two fuzzy sets, their intersection, union, and complement are computed as follows:

1. (*M*∩*N*)(*x*)=*M*(*x*)∧*N*(*x*)=min(*M*(*x*), *N*(*x*))
2. (*M* ∪ *N*)(*x*)=*M*(*x*)∨*N*(*x*)=max(*M*(*x*), *N*(*x*))
3. *M* ^*c*^(*x*)=1 − *M*(*x*)

If *R*_*B*_ satisfies,

1. reflexivity: *R*_*B*_(*x*, *x*)=1, ∀*x* ∈ *U*
2. symmetry: *R*_*B*_(*x*, *y*)=*R*_*B*_(*y*, *x*), ∀*x*, *y* ∈ *U*

then, *R*_*B*_ is the fuzzy similarity relation on *U*. For ∀*x*, *y* ∈ *U*, the fuzzy neighborhood of *x* about *R*_*B*_ can be expressed as [*x*]_*R*_*B*__(*y*)=*R*_*B*_(*x*, *y*), *y* ∈ *U*, which is a fuzzy set on *U*.

### 2.3. Fuzzy Neighborhood Granule

The fuzzy relation can accurately describe the relationship between samples, but the strength of association between samples is different. To reduce the influence of redundant and noise in the data, the neighborhood radius can be set to filter out some weak correlation data to improve the computational efficiency. Wang et al. [20] proposed the concept of fuzzy neighborhood:

Let 〈*U*, *A*, *D*〉 be a decision information system. For *U*={*x*_1_, *x*_2_,…, *x*_*n*_}, *B*⊆*A*, ∀*x*, *y* ∈ *U*, *R*_*B*_ is the fuzzy similarity relation on *U*. For ∀*b* ∈ *B*, the fuzzy neighborhood of *x* with respect to *R*_*B*_ is defined as

(1) $$\begin{array}{c} {\left[x\right]}_{R_{B}}\left(y\right)=R_{B}\left(x,y\right)=\underset{b\in B}{\text{min}}R_{b}\left(x,y\right), \end{array}$$

where *R*_*b*_(*x*, *y*)=1 − |*x*_*b*_ − *y*_*b*_| and *x*_*b*_ and *y*_*b*_, respectively, represent the value of the corresponding attribute *b*. For ∀*B* ∈ *A*, the fuzzy neighborhood granule of *x* with respect to *R*_*B*_ is defined as

(2) $$\begin{array}{c} {\left[x\right]}_{R_{B}}^{\alpha }\left(y\right)=\left\{\begin{array}{cc} R_{B}\left(x,y\right), & R_{B}\left(x,y\right)\geq \alpha ,\\ 0, & R_{B}\left(x,y\right)<\alpha , \end{array}\right. \end{array}$$

where *α* is the fuzzy neighborhood radius, and when *α*_1_ ≥ *α*_2_, [*x*]_*R*_*B*__^*α*_1_^(*y*)⊆[*x*]_*R*_*B*__^*α*_2_^(*y*).

## 3. Rough Uncertainty Metric

In this section, the paper introduces the rough decision by using the fuzzy similarity relation between samples to further determine the inclusion of equivalence classes accurately. Combining the rough decision with the fuzzy neighborhood granule of the sample, the definition of the conditional entropy with respect to the attribute set and the proof of its related theorems are given. Then, the rough uncertainty metric model is proposed, and the corresponding feature genes selection algorithm is designed according to the proposed model.

### 3.1. Rough Decision

Generally, the decision equivalence classes of the general domain are divided by decision attributes, but there is always a correlation between samples. For example, a fuzzy neighborhood granule of a sample contains samples with different equivalent decision classes. Therefore, a sample cannot be completely categorized into a decision equivalence class. In this paper, the fuzzy similarity relation is used to calculate the membership degree of each sample for different decision equivalence classes, and a more accurate rough decision is proposed.

**Definition 1 .**

Let 〈*U*, *A*, *D*〉 be a decision information system. *U*={*x*_1_, *x*_2_,…, *x*_*n*_}; *U*/*D*={*D*_1_, *D*_2_,…, *D*_*r*_}, *R*_*A*_ is the fuzzy similarity relation on *U*, and the rough decision RD is defined as follows:

(3) $$\begin{array}{c} \text{RD}=\left\{{\text{RD}}_{1}^{T},{\text{RD}}_{2}^{T},\ldots ,{\text{RD}}_{r}^{T}\right\},\\ {\text{RD}}_{j}=\left\{{\text{RD}}_{j}\left(x_{1}\right),{\text{RD}}_{j}\left(x_{2}\right),\ldots ,{\text{RD}}_{j}\left(x_{n}\right)\right\},\\ {\text{RD}}_{j}\left(x_{i}\right)=\frac{\sum {\left[x_{i}\right]}_{R_{A}}\left(d\right)}{\sum {\left[x_{i}\right]}_{R_{A}}\left(y\right)}, \end{array}$$

where *d* ∈ *D*_*j*_, *i*=1,2,…, *n*, and *j*=1,2,…, *r*.

### 3.2. Rough Uncertainty Metric Model

In the data characterization stage, the original information of the data is restored as much as possible. Next, fuzzy neighborhood granule and rough decision are used to realize the uncertainty metric of feature genes, and the evaluation method of the significance of feature genes is given.

**Definition 2 .**

Let 〈*U*, *A*, *D*〉 be a decision information system. ∀*B*, *C*⊆*A* are two conditional attribute subsets, and [*x*]_*R*_*B*__^*α*^(*y*) is the fuzzy neighborhood granule of *x* with radius *α*. The rough entropy with respect to *B* is defined as

(4) $$\begin{array}{c} E_{\text{c}}\left(B\right)=-\frac{1}{\left|U\right|}\overset{\left|U\right|}{\underset{i=1}{\sum }}{\text{log}}_{2}\frac{1}{\left|{\left[x_{i}\right]}_{R_{B}}^{\alpha }\left(y\right)\right|}. \end{array}$$

The joint entropy with respect to *B*, *C* is defined as

(5) $$\begin{array}{c} E_{\text{c}}\left(B,C\right)=-\frac{1}{\left|U\right|}\overset{\left|U\right|}{\underset{i=1}{\sum }}{\text{log}}_{2}\frac{1}{\left|{\left[x_{i}\right]}_{R_{B}}^{\alpha }\left(y\right)\cap {\left[x_{i}\right]}_{R_{C}}^{\alpha }\left(y\right)\right|}, \end{array}$$

where ∀*x*, *y* ∈ *U*, |[*x*_*i*_]_*R*_*B*__^*α*^(*y*)| represents the number of nonzero values in the fuzzy neighborhood granule of the object *x*_*i*_, and 1/|[*x*_*i*_]_*R*_*B*__^*α*^(*y*)| represents the probability of an element in the fuzzy neighborhood granule |[*x*_*i*_]_*R*_*B*__^*α*^(*y*)|.

**Definition 3 .**

Let *S* and *T* be two fuzzy sets. |*S*∩*T*| is defined as the number of nonzero values of objects whose membership degree in *S* is not greater than that of *T*.

**Example 1 .**

Given a sample set *U*={*x*_1_, *x*_2_,…, *x*_10_}, *S* and *T* are two fuzzy sets on *U*, the membership of the samples on them are assumed as follows:

(6) $$\begin{array}{c} S=\left\{\frac{0.7}{x_{1}},\frac{0.4}{x_{2}},\frac{0.5}{x_{3}},\frac{0.7}{x_{4}},\frac{0.8}{x_{5}},\frac{0.3}{x_{6}},\frac{0.5}{x_{7}},\frac{0.1}{x_{8}},\frac{0.2}{x_{9}},\frac{0.6}{x_{10}}\right\},\\ T=\left\{\frac{0.5}{x_{1}},\frac{0.4}{x_{2}},\frac{0.3}{x_{3}},\frac{0.6}{x_{4}},\frac{0.4}{x_{5}},\frac{0.7}{x_{6}},\frac{0.4}{x_{7}},\frac{0.9}{x_{8}},\frac{0.2}{x_{9}},\frac{0.3}{x_{10}}\right\}. \end{array}$$

|*S*∩*T*| represents the number of nonzero values of objects whose membership degree in *S* is not greater than that of *T*. Thus,

(7) $$\begin{array}{c} \left|S\cap T\right|=\left|\left\{x_{2},x_{6},x_{8},x_{9}\right\}\right|=4. \end{array}$$

Similarly, |*T*∩*S*| represents the number of nonzero values of objects whose membership degree in *T* is not greater than that of *S*:

(8) $$\begin{array}{c} \left|T\cap S\right|=\left|\left\{x_{1},x_{2},x_{3},x_{4},x_{5},x_{7},x_{9},x_{10}\right\}\right|=8. \end{array}$$

**Definition 4 .**

Let 〈*U*, *A*, *D*〉 be a decision information system. *U*/*D*={*D*_1_, *D*_2_,…, *D*_*r*_}, *B*⊆*A* is a conditional attribute subset, fuzzy neighborhood granule [*x*]_*R*_*B*__^*α*^(*y*), and rough decision RD are two fuzzy matrices. Then, the conditional entropy of the decision attribute set *D* with respect to *B* on *U* is defined as

(9) $$\begin{array}{c} E_{\text{c}}\left(\text{RD}\;|\;B\right)=\frac{1}{\left|U\right|}\overset{\left|U\right|}{\underset{i=1}{\sum }}{\text{log}}_{2}\frac{\left|{\left[x_{i}\right]}_{R_{B}}^{\alpha }\left(y\right)\right|}{\left|{\left[x_{i}\right]}_{R_{B}}^{\alpha }\left(y\right)\cap {\left[x_{i}\right]}_{\text{RD}}\left(y\right)\right|}, \end{array}$$

where [*x*_*i*_]_RD_(*y*) represents the rough decision RD_*r*_^*T*^ corresponding to the equivalence class *D*_*r*_ to which the sample *x*_*i*_ belongs.

**Example 2 .**

Given a decision table 〈*U*, *A*, *D*〉, where *U*={*x*_1_, *x*_2_,…, *x*_5_}, *U*/*D*={*D*_1_, *D*_2_}, *D*_1_={*x*_1_, *x*_2_, *x*_3_}, and *D*_2_={*x*_4_, *x*_5_}, [*x*]_*R*_*A*__(*y*) is the fuzzy similarity relation matrix with respect to attribute set *A* and

(10) $$\begin{array}{c} {\left[x\right]}_{R_{A}}\left(y\right)=\left[\begin{array}{ccc} R_{A}\left(x_{1},x_{1}\right) & \ldots & R_{A}\left(x_{1},x_{5}\right)\\ \vdots & \ddots & \vdots \\ R_{A}\left(x_{5},x_{1}\right) & \cdots & R_{A}\left(x_{5},x_{5}\right) \end{array}\right]\\ =\left[\begin{array}{ccccc} 1 & 0.53 & 0.7 & 0 & 0\\ 0.53 & 1 & 0.7 & 0.2 & 0.62\\ 0.7 & 0.7 & 1 & 0.11 & 0.5\\ 0 & 0.2 & 0.11 & 1 & 0.7\\ 0 & 0.62 & 0.5 & 0.7 & 1 \end{array}\right]. \end{array}$$

According to the given conditions and Definition 1,

(11) $$\begin{array}{c} {\text{RD}}_{1}\left(x_{1}\right)=\frac{\sum {\left[x_{1}\right]}_{R_{A}}\left(d\right)}{\sum {\left[x_{1}\right]}_{R_{A}}\left(y\right)}=\frac{1+0.53+0.7}{1+0.53+0.7+0+0}=1,\\ {\text{RD}}_{1}\left(x_{2}\right)=\frac{\sum {\left[x_{2}\right]}_{R_{A}}\left(d\right)}{\sum {\left[x_{2}\right]}_{R_{A}}\left(y\right)}=\frac{0.53+1+0.7}{0.53+1+0.7+0.2+0.62}=0.7311,\\ {\text{RD}}_{1}\left(x_{3}\right)=\frac{\sum {\left[x_{3}\right]}_{R_{A}}\left(d\right)}{\sum {\left[x_{3}\right]}_{R_{A}}\left(y\right)}=\frac{0.7+0.7+1}{0.7+0.7+1+0.11+0.5}=0.7973,\\ {\text{RD}}_{1}\left(x_{4}\right)=\frac{\sum {\left[x_{4}\right]}_{R_{A}}\left(d\right)}{\sum {\left[x_{4}\right]}_{R_{A}}\left(y\right)}=\frac{0+0.2+0.11}{0+0.2+0.11+1+0.7}=0.1542,\\ {\text{RD}}_{1}\left(x_{5}\right)=\frac{\sum {\left[x_{5}\right]}_{R_{A}}\left(d\right)}{\sum {\left[x_{5}\right]}_{R_{A}}\left(y\right)}=\frac{0+0.62+0.5}{0+0.62+0.5+0.7+1}=0.3972. \end{array}$$

Then, RD_1_={1,0.7311, 0.7973, 0.1542, 0.3972}.

Similarly,

(12) $$\begin{array}{c} {\text{RD}}_{2}\left(x_{1}\right)=\frac{\sum {\left[x_{1}\right]}_{R_{A}}\left(d\right)}{\sum {\left[x_{1}\right]}_{R_{A}}\left(y\right)}=\frac{0+0}{1+0.53+0.7+0+0}=0,\\ {\text{RD}}_{2}\left(x_{2}\right)=\frac{\sum {\left[x_{2}\right]}_{R_{A}}\left(d\right)}{\sum {\left[x_{2}\right]}_{R_{A}}\left(y\right)}=\frac{0.2+0.62}{0.53+1+0.7+0.2+0.62}=0.2689,\\ {\text{RD}}_{2}\left(x_{3}\right)=\frac{\sum {\left[x_{3}\right]}_{R_{A}}\left(d\right)}{\sum {\left[x_{3}\right]}_{R_{A}}\left(y\right)}=\frac{0.11+0.5}{0.7+0.7+1+0.11+0.5}=0.2027,\\ {\text{RD}}_{2}\left(x_{4}\right)=\frac{\sum {\left[x_{4}\right]}_{R_{A}}\left(d\right)}{\sum {\left[x_{4}\right]}_{R_{A}}\left(y\right)}=\frac{1+0.7}{0+0.2+0.11+1+0.7}=0.8458,\\ {\text{RD}}_{2}\left(x_{5}\right)=\frac{\sum {\left[x_{5}\right]}_{R_{A}}\left(d\right)}{\sum {\left[x_{5}\right]}_{R_{A}}\left(y\right)}=\frac{0.7+1}{0+0.62+0.5+0.7+1}=0.6028. \end{array}$$

Then, RD_1_={0,0.2689, 0.2027, 0.8458, 0.0.6028}.

So, the rough decision is as follows:

(13) $$\begin{array}{c} \text{RD}=\left[\begin{array}{cc} 1 & 0\\ 0.7311 & 0.2689\\ 0.7973 & 0.2027\\ 0.1542 & 0.8458\\ 0.3972 & 0.6028 \end{array}\right]. \end{array}$$

Here, the neighborhood radius *α*=0 (the specific experiment will discuss the parameters according to different data sets). According to Definition 2, |[*x*_*i*_]_*R*_*A*__^*α*^(*y*)| represents the number of nonzero values in the fuzzy neighborhood granule of the object *x*_*i*_, and then,

(14) $$\begin{array}{c} \left|{\left[x_{1}\right]}_{R_{A}}^{\alpha }\left(y\right)\right|=\left|\left\{1,0.53,0.7,0,0\right\}\right|=3,\\ \left|{\left[x_{2}\right]}_{R_{A}}^{\alpha }\left(y\right)\right|=\left|\left\{0.53,1,0.7,0.2,0.62\right\}\right|=5,\\ \left|{\left[x_{3}\right]}_{R_{A}}^{\alpha }\left(y\right)\right|=\left|\left\{0.7,0.7,1,0.11,0.5\right\}\right|=5,\\ \left|{\left[x_{4}\right]}_{R_{A}}^{\alpha }\left(y\right)\right|=\left|\left\{0,0.2,0.11,1,0.7\right\}\right|=4,\\ \left|{\left[x_{5}\right]}_{R_{A}}^{\alpha }\left(y\right)\right|=\left|\left\{0,0.62,0.5,0.7,1\right\}\right|=4, \end{array}$$

where [*x*_*i*_]_RD_(*y*) represents the rough decision RD_*r*_^*T*^ corresponding to the equivalence class *D*_*r*_ to which the sample *x*_*i*_ belongs, where *r*=1,2. Since *x*_1_, *x*_2_, *x*_3_ ∈ *D*_1_ and *x*_4_, *x*_5_ ∈ *D*_2_,

(15) $$\begin{array}{c} {\left[x_{1}\right]}_{\text{RD}}\left(y\right)=\left\{1,0.7311,0.7973,0.1542,0.3972\right\},\\ {\left[x_{2}\right]}_{\text{RD}}\left(y\right)=\left\{1,0.7311,0.7973,0.1542,0.3972\right\},\\ {\left[x_{3}\right]}_{\text{RD}}\left(y\right)=\left\{1,0.7311,0.7973,0.1542,0.3972\right\},\\ {\left[x_{4}\right]}_{\text{RD}}\left(y\right)=\left\{0,0.2689,0.2027,0.8458,0.6028\right\},\\ {\left[x_{5}\right]}_{\text{RD}}\left(y\right)=\left\{0,0.2689,0.2027,0.8458,0.6028\right\}. \end{array}$$

According to Definition 3, [*x*_*i*_]_*R*_*A*__^*α*^(*y*) is similar to *S* and [*x*_*i*_]_RD_(*y*) is similar to *T* in it. Then,

(16) $$\begin{array}{c} \left|{\left[x_{1}\right]}_{R_{A}}^{\alpha }\left(y\right)\cap {\left[x_{1}\right]}_{\text{RD}}\left(y\right)\right|=3,\\ \left|{\left[x_{2}\right]}_{R_{A}}^{\alpha }\left(y\right)\cap {\left[x_{2}\right]}_{\text{RD}}\left(y\right)\right|=2,\\ \left|{\left[x_{3}\right]}_{R_{A}}^{\alpha }\left(y\right)\cap {\left[x_{3}\right]}_{\text{RD}}\left(y\right)\right|=3,\\ \left|{\left[x_{4}\right]}_{R_{A}}^{\alpha }\left(y\right)\cap {\left[x_{4}\right]}_{\text{RD}}\left(y\right)\right|=2,\\ \left|{\left[x_{5}\right]}_{R_{A}}^{\alpha }\left(y\right)\cap {\left[x_{5}\right]}_{\text{RD}}\left(y\right)\right|=1. \end{array}$$

The remaining samples are equally available. Therefore, the conditional entropy of the decision attribute set *D* with respect to *A* on *U* is

(17) $$\begin{array}{c} E_{\text{c}}\left(\text{RD}\;|\;A\right)=\frac{1}{\left|U\right|}\overset{\left|U\right|}{\underset{i=1}{\sum }}{\text{log}}_{2}\frac{\left|{\left[x_{i}\right]}_{R_{A}}^{\alpha }\left(y\right)\right|}{\left|{\left[x_{i}\right]}_{R_{A}}^{\alpha }\left(y\right)\cap {\left[x_{i}\right]}_{\text{RD}}\left(y\right)\right|}\\ =\frac{1}{5}\overset{5}{\underset{i=1}{\sum }}{\text{log}}_{2}\frac{\left|{\left[x_{i}\right]}_{R_{A}}^{\alpha }\left(y\right)\right|}{\left|{\left[x_{i}\right]}_{R_{A}}^{\alpha }\left(y\right)\cap {\left[x_{i}\right]}_{\text{RD}}\left(y\right)\right|}\\ =\frac{1}{5}\left({\text{log}}_{2}\frac{3}{3}+\;\;{\text{log}}_{2}\frac{5}{2}+\;\;{\text{log}}_{2}\frac{5}{3}+\;\;{\text{log}}_{2}\frac{4}{2}+\;\;{\text{log}}_{2}\frac{4}{1}\right)\\ =1.0118. \end{array}$$

**Theorem 1 .**

Let 〈*U*, *A*, *D*〉 be a decision information system, *U*/*D*={[*x*_1_]_*D*_, [*x*_2_]_*D*_,…, [*x*_|*U*|_]_*D*_}, *B*⊆*A* is a conditional attribute subset, *RD* is a rough decision defined on decision attribute set *D*. Then,

(18) $$\begin{array}{c} E_{\text{c}}\left(\text{RD}\;|\;B\right)=E_{\text{c}}\left(B\right)-E_{\text{c}}\left(\text{RD},B\right). \end{array}$$

**Proof**

According to Definitions 4 and 2, the derivation process is as follows:

(19) $$\begin{array}{c} E_{\text{c}}\left(\text{RD}\;|\;B\right)=\frac{1}{\left|U\right|}\overset{\left|U\right|}{\underset{i=1}{\sum }}{\text{log}}_{2}\frac{\left|{\left[x_{i}\right]}_{R_{B}}^{\alpha }\left(y\right)\right|}{\left|{\left[x_{i}\right]}_{R_{B}}^{\alpha }\left(y\right)\cap {\left[x_{i}\right]}_{\text{RD}}\left(y\right)\right|}\\ =\frac{1}{\left|U\right|}\overset{\left|U\right|}{\underset{i=1}{\sum }}\left({\text{log}}_{2}\left|{\left[x_{i}\right]}_{R_{B}}^{\alpha }\left(y\right)\right|-\;\;{\text{log}}_{2}\left|{\left[x_{i}\right]}_{R_{B}}^{\alpha }\left(y\right)\cap {\left[x_{i}\right]}_{\text{RD}}\left(y\right)\right|\right)\\ =-\frac{1}{\left|U\right|}\overset{\left|U\right|}{\underset{i=1}{\sum }}{\text{log}}_{2}\frac{1}{\left|{\left[x_{i}\right]}_{R_{B}}^{\alpha }\left(y\right)\right|}-\left(-\frac{1}{\left|U\right|}\overset{\left|U\right|}{\underset{i=1}{\sum }}{\text{log}}_{2}\frac{1}{\left|{\left[x_{i}\right]}_{R_{B}}^{\alpha }\left(y\right)\cap {\left[x_{i}\right]}_{\text{RD}}\left(y\right)\right|}\right)\\ =E_{c}\left(B\right)-E_{c}\left(\text{RD},B\right). \end{array}$$

Hence, *E*_c_(RD | *B*)=*E*_c_(*B*) − *E*_c_(RD, *B*).

**Theorem 2 .**

Let 〈*U*, *A*, *D*〉 be a decision information system, ∀*B*⊆*A*, *E*_c_(RD | *B*) ≥ 0.

**Proof**

Assume *E*_c_(RD | *B*) < 0 equivalent to log_2_(|[*x*_*i*_]_*R*_*B*__^*α*^(*y*)|/|[*x*_*i*_]_*R*_*B*__^*α*^(*y*)∩[*x*_*i*_]_RD_(*y*)|) < 0, that is, |[*x*_*i*_]_*R*_*B*__^*α*^(*y*)| < |[*x*_*i*_]_*R*_*B*__^*α*^(*y*)∩[*x*_*i*_]_RD_(*y*)|, obviously not established. So ∀*B*⊆*A*, *E*_c_(RD | *B*) ≥ 0.

**Theorem 3 .**

Let 〈*U*, *A*, *D*〉 be a decision information system, ∀*M*, *N*⊆*A* are two conditional attribute subsets. If [*x*]_*R*_*M*__^*α*^(*y*)⊆[*x*]_*R*_*N*__^*α*^(*y*), then *E*_c_(*RD* | *M*) ≤ *E*_c_(*RD* | *N*); the equation is true if and only if [*x*]_*R*_*M*__^*α*^(*y*)=[*x*]_*R*_*N*__^*α*^(*y*).

**Corollary 1 .**

If *N*⊆*M*, then *E*_c_(*RD* | *M*) ≤ *E*_c_(*RD* | *N*).

**Proof**

(20) $$\begin{array}{c} E_{\text{c}}\left(\text{RD}\;|\;N\right)-E_{\text{c}}\left(\text{RD}\;|\;M\right)=\frac{1}{\left|U\right|}\overset{\left|U\right|}{\underset{i=1}{\sum }}{\text{log}}_{2}\frac{\left|{\left[x_{i}\right]}_{R_{N}}^{\alpha }\left(y\right)\right|}{\left|{\left[x_{i}\right]}_{R_{N}}^{\alpha }\left(y\right)\cap {\left[x_{i}\right]}_{\text{RD}}\left(y\right)\right|}-\frac{1}{\left|U\right|}\overset{\left|U\right|}{\underset{i=1}{\sum }}{\text{log}}_{2}\frac{\left|{\left[x_{i}\right]}_{R_{M}}^{\alpha }\left(y\right)\right|}{\left|{\left[x_{i}\right]}_{R_{M}}^{\alpha }\left(y\right)\cap {\left[x_{i}\right]}_{\text{RD}}\left(y\right)\right|}\\ =\frac{1}{\left|U\right|}\overset{\left|U\right|}{\underset{i=1}{\sum }}\left({\text{log}}_{2}\frac{\left|{\left[x_{i}\right]}_{R_{N}}^{\alpha }\left(y\right)\right|}{\left|{\left[x_{i}\right]}_{R_{N}}^{\alpha }\left(y\right)\cap {\left[x_{i}\right]}_{\text{RD}}\left(y\right)\right|}-\;\;{\text{log}}_{2}\frac{\left|{\left[x_{i}\right]}_{R_{M}}^{\alpha }\left(y\right)\right|}{\left|{\left[x_{i}\right]}_{R_{M}}^{\alpha }\left(y\right)\cap {\left[x_{i}\right]}_{\text{RD}}\left(y\right)\right|}\right)\\ =\frac{1}{\left|U\right|}\overset{\left|U\right|}{\underset{i=1}{\sum }}{\text{log}}_{2}\frac{\left|{\left[x_{i}\right]}_{R_{N}}^{\alpha }\left(y\right)\right|\cdot \left|{\left[x_{i}\right]}_{R_{M}}^{\alpha }\left(y\right)\cap {\left[x_{i}\right]}_{\text{RD}}\left(y\right)\right|}{\left|{\left[x_{i}\right]}_{R_{N}}^{\alpha }\left(y\right)\cap {\left[x_{i}\right]}_{\text{RD}}\left(y\right)\right|\cdot \left|{\left[x_{i}\right]}_{R_{M}}^{\alpha }\left(y\right)\right|}. \end{array}$$

According to the definition of the fuzzy neighborhood granule, if *N*⊆*M*, [*x*]_*R*_*M*__^*α*^(*y*)⊆[*x*]_*R*_*N*__^*α*^(*y*), combined with Definition 3, |[*x*_*i*_]_*R*_*N*__^*α*^(*y*)∩[*x*_*i*_]_RD_(*y*)| · |[*x*_*i*_]_*R*_*M*__^*α*^(*y*)| ≤ |[*x*_*i*_]_*R*_*N*__^*α*^(*y*)| · |[*x*_*i*_]_*R*_*M*__^*α*^(*y*)∩[*x*_*i*_]_RD_(*y*)|, *E*_c_(RD | *N*) − *E*_c_(RD | *M*) ≥ 0, that is, *E*_c_(RD | *M*) ≤ *E*_c_(RD | *N*).

**Theorem 4 .**

Let 〈*U*, *A*, *D*〉 be a decision information system, *B*⊆*A*. ∀*b* ∈ *B*, if *E*_c_(*RD* | *B* − {*b*})=*E*_c_(*RD* | *B*), then the attribute *b* is unnecessary.

**Proof**

Assume ∃*b*⊆*B* is necessary and satisfies *E*_c_(RD | *B* − {*b*})=*E*_c_(RD | *B*). According to the definition of fuzzy neighborhood granule, if the attribute *b* is necessary, then [*x*]_*R*_*B*−{*b*}__^*α*^(*y*) ≠ [*x*]_*R*_*B*__^*α*^(*y*). And *B* − {*b*}⊆*B*, then by Theorem 3 and Corollary 1, *E*_c_(RD | *B* − {*b*}) > *E*_c_(RD | *B*), obviously not in line with the assumption. So ∀*b* ∈ *B*, if *E*_c_(RD | *B* − {*b*})=*E*_c_(RD | *B*), then the attribute *b* is unnecessary.

Generally, given a decision information system 〈*U*, *A*, *D*〉, *B*⊆*A* is conditional attribute subset. Let *B* be a reduction of *A*, if *B* satisfies the following conditions:

1. *E*_c_(RD | *B*)=*E*_c_(RD | *A*)
2. ∀*b* ∈ *B*, *E*_c_(RD | *B* − {*b*}) > *E*_c_(RD | *B*).

Due to the inconsistency and noises in the data sets, it becomes difficult to find the smallest accurate reduction [31]. Therefore, this paper employs the variable precision model to tolerate the error between the conditional entropy of the reduction attribute subset and the conditional entropy of the original attribute set and set the parameter *β* as constraint. That is, if red satisfies the condition *E*_c_(RD | red) − *E*_c_(RD | *A*) ≤ *β*, red can be used as a reduction of *A*.

**Definition 5 .**

Let 〈*U*, *A*, *D*〉 be a decision information system, ∀*a* ∈ *A*, the significance of condition attribute *a* relative to *A* is defined as

(21) $$\begin{array}{c} \text{SIG}\left(a,A,D\right)=E_{\text{c}}\left(\text{RD}\;|\;A-\left\{a\right\}\right)-E_{\text{c}}\left(\text{RD}\;|\;A\right). \end{array}$$

∀*r* ∈ *A* − red, the significance of condition attribute *r* relative to red is defined as

(22) $$\begin{array}{c} \text{SIG}\left(r,\text{red},D\right)=E_{\text{c}}\left(\text{RD}\;|\;\text{red}\right)-E_{\text{c}}\left(\text{RD}\;|\;\text{red}\cup \left\{r\right\}\right). \end{array}$$

### 3.3. Feature Genes Selection Algorithm Based on Rough Uncertainty Metric Model

For the above theory, this paper designs a feature genes selection algorithm based on the rough uncertainty metric model. As shown in Algorithm 1, the application of the new model in feature genes selection is realized.

## 4. Experimental Results and Analysis

In addition to the richness of the theory, a good model needs to have a good practical effect. Therefore, the experimental contrast analysis is set up in this part. Under the same conditions, the same genetic data are used to compare our algorithm with other existing similar algorithms. The specific experimental results data are used to illustrate the advantages of the proposed model.

### 4.1. Experiment Preparation

In order to verify the validity of the proposed model, four data sets are selected from the public data sources as experimental objects. The specific information is shown in Table 1. The data sets WPBC, WDBC, and Heart-Cle are selected from the UCI Machine Learning Repository, and Colon is selected from http://datam.i2r.a-star.edu.sg/datasets/krbd/. At the beginning of the data processing stage, in order to eliminate the influence of value dimension inconsistency among features, all numerical experimental data will be normalized and mapped to [0, 1] by the formula *f*(*x*_*i*_)=(*x*_*i*_ − *x*_min_)(*x*_max_ − *x*_min_). In addition, the fuzzy similarity relation of samples *x*_*i*_ and *x*_*j*_ with respect to an attribute is calculated as

(23) $$\begin{array}{c} r_{ij}=\left\{\begin{array}{cc} 1-\left|x_{i}-x_{j}\right|, & \left|x_{i}-x_{j}\right|\leq 1-\alpha ,\\ 0, & \left|x_{i}-x_{j}\right|>1-\alpha . \end{array}\right. \end{array}$$

### 4.2. Parameter Discussion

In order to reduce the influence of noise and redundancy in the data used in the experiment, this paper sets the parameter *α* as the neighborhood radius to calculate the fuzzy neighborhood granule of the sample, which will filter out the data that are less relevant to the sample and consumes computation time as much as possible, improving the efficiency of the experiment to some extent. Due to the objective conditions in reality, these noise and redundant data can only be minimized but cannot be completely avoided. Therefore, the experimental results need to tolerate the error effects caused by these data. So, the parameter *β* is set in this paper to control the size of the error in the experiment; thus, a smaller feature genes subset with higher classification accuracy is selected. Since different data sets have different correlation strengths, the parameters need to be set separately according to different data sets.

**Figure 1:**
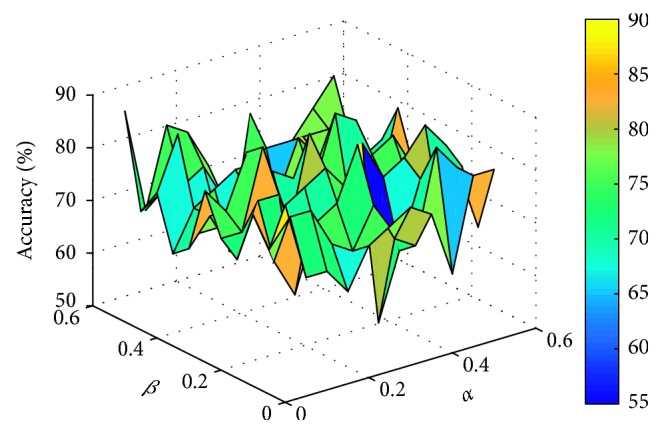
Accuracy varying with parameters *α* and *β* (WPBC).

**Figure 2 fig2:**
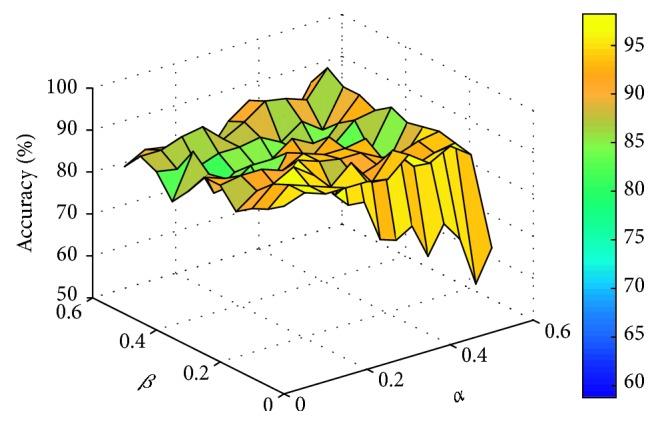
Accuracy varying with parameters *α* and *β* (WDBC).

**Figure 3 fig3:**
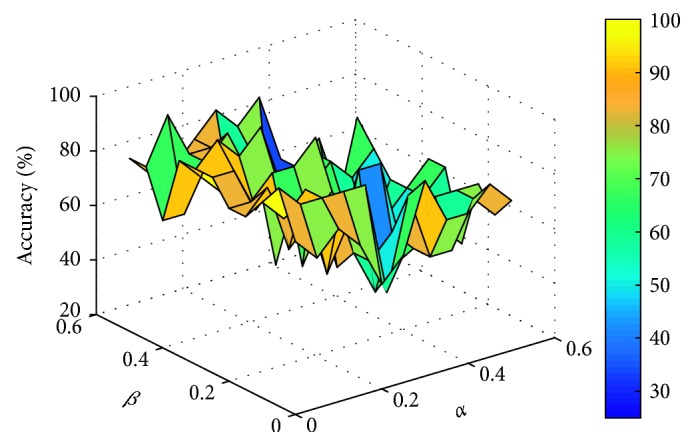
Accuracy varying with parameters *α* and *β* (Colon).

**Figure 4:**
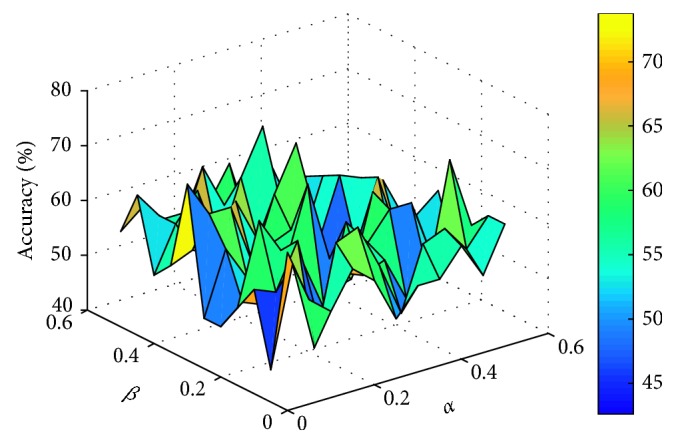
Accuracy varying with parameters *α* and *β* (Heart-Cle).

This paper sets the parameters *α* and *β* to vary from 0 to 0.5, respectively, with an interval of 0.05. For different data sets, the experiment compares the number of feature genes and the corresponding classification accuracy obtained by different parameters. Two classifiers, support vector machine (linear SVM) and K-nearest neighbor (KNN, *K* = 3), are used to evaluate the classification accuracy of feature genes subset by 10-fold cross-validation. The comparison process is shown in Figures 1 [Fig fig2] [Fig fig3]–4 (the classification accuracy in the figures is based on the linear-SVM classifier, which is the same as the experimental result under the 3NN classifier). Finally, the selected feature genes subset and corresponding suitable parameters under different data sets are shown in Table 2.

### 4.3. Experimental Comparison

In this section, in order to verify the validity of the proposed model, the designed algorithm is compared with the existing similar algorithms. The experimental objects include original data without algorithm processing (raw data), feature selection algorithm based on fuzzy entropy (FISEN) [32], feature selection algorithm based on fuzzy neighborhood rough set (FNRS) [19], and algorithm proposed in this paper (RUM). The comparison includes two aspects: the number of selected feature genes and the classification accuracy.

A good feature genes selection algorithm aims at finding a subset of smaller feature genes that make classification more accurate. First, the number of original data and selected feature genes under different algorithms are shown in Table 3. It is not difficult to find from the table that the number of feature genes in the original data is more, and significant reduction is achieved by different algorithms. Comparing the average number of feature genes, the FNRS algorithm is lower than the FISEN algorithm, and the RUM algorithm is the least among all the compared algorithms. In different data sets, the RUM algorithm is also the least compared to other comparison algorithms. Therefore, the RUM algorithm proposed in this paper is superior in terms of the number of selected feature genes.

Certainly, only relying on the minimum number of selected feature genes is not enough to illustrate the advantages of an algorithm. If the experimental results can achieve equal or even higher classification accuracy on a relatively small subset of feature genes, the algorithm can be proved to be excellent.  Table 4 shows the classification accuracy of the original data and the data obtained from the different algorithms under classifier linear-SVM. In general, the RUM algorithm proposed in this paper is the highest among the four experimental objects in terms of average classification accuracy. From the perspective of a single data set, the two classification accuracies obtained by the RUM algorithm and the FNRS algorithm are the same on the WDBC data set, which are higher than others, and the same on the Colon data set. On the remaining data sets, the classification accuracy obtained by the RUM algorithm is the highest, especially on the WPBC data set, which is about 7-8 percent higher than the FNRS algorithm.

Comparing the classification accuracy on a classifier alone, the persuasive power may be insufficient. In this paper, the classifier 3NN is added to further verify the advantages of the proposed RUM algorithm and is shown in Table 5. First, the average classification accuracy obtained by the RUM algorithm is the highest. Apparently, the classification accuracy of the RUM algorithm on the WDBC data set is slightly lower than that of the original data and the FNRS algorithm, but this is not enough to show that the RUM algorithm is not good. It can be seen from Table 3 that the number of feature genes obtained by the RUM algorithm on the WDBC data set is the least, even less than one-third of the FNRS algorithm and the FISEN algorithm, which is only one-sixth of the original data. In the meantime, the classification accuracy achieved by the RUM algorithm is only less than 1 percent lower than the original data and FNRS algorithm, and higher than the FISEN algorithm. In addition, on the other three data sets, data selected by the RUM algorithm has the highest classification accuracy, especially on the WPBC and Colon data sets, which is about 10 percent higher than other algorithms.

From the above concept, the advantage of the rough uncertainty metric model in the feature genes selection has been well verified by comparing the number of selected feature genes and analyzing the classification accuracy on the two classifiers.

## 5. Conclusions and Future Works

A novel model is established in this paper. In this model, the fuzzy neighborhood granule of the sample are constructed by combining the fuzzy concept with the neighborhood concept, and the decision equivalence class is further accurately expressed as rough decision by using the fuzzy similarity relation between samples. The original information between the data is reserved as perfectly as possible during the data characterization phase. Then fuzzy neighborhood granule and rough decision are introduced into conditional entropy, and a metric method is proposed to evaluate the significance of feature genes. Based on the nature of this model, which is proved by the four theorems in the paper, a feature gene selection algorithm is designed. Finally, the proposed algorithm is compared with the existing similar algorithms on the common data sets. The experimental results show that the proposed algorithm can obtain a relatively small subset of feature genes and achieve better classification results, which verify the validity of the proposed model. However, this model still has something inadequate: the selected parameters based on the corresponding single data set are not generalized, requiring further study and improvement in the future work. In the next step, our study will focus on the parameter selection problem and find out how to adaptively set the appropriate generalized parameters.

## Figures and Tables

**Algorithm 1:**
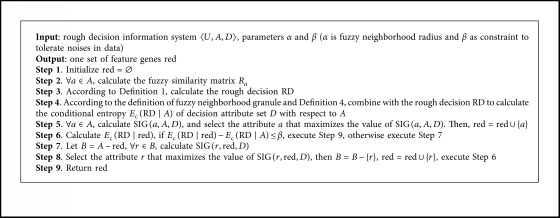
Feature genes selection based on the rough uncertainty metric.

**Table 1:** Description of data sets.

Data sets	Sample	Attributes	Classes
WPBC	198	32	2
WDBC	569	30	2
Colon	62	2000	2
Heart-Cle	303	13	5

**Table 2:** Suitable parameters and selected feature genes of different data sets.

Data sets	Values of *α*	Values of *β*	Selected feature genes
WPBC	0.1	0.25	1, 32, 24
WDBC	0.1	0.1	28, 22, 1, 25, 7
Colon	0.1	0.3	1895, 267, 897, 1990
Heart-Cle	0.35	0.35	9, 12, 11, 7, 13

**Table 3:** Number of selected feature genes under different algorithms.

Data Sets	Raw data	FISEN	FNRS	RUM
WPBC	32	16	8	3
WDBC	30	16	18	5
Colon	2000	10	5	4
Heart-Cle	13	11	7	5
Average	518.75	13.25	9.50	4.25

**Table 4:** Classification accuracy of selected data under linear-SVM.

Data Sets	Raw data	FISEN	FNRS	RUM
WPBC	74.24	81.82	82.50	**90.00**
WDBC	93.16	95.26	**97.37**	**97.37**
Colon	75.00	83.33	**92.31**	**92.31**
Heart-Cle	59.41	56.44	65.57	**70.49**
Average	75.45	79.21	84.44	**87.54**

**Table 5:** Classification accuracy of selected data under 3NN.

Data Sets	Raw data	FISEN	FNRS	FNCE
WPBC	74.84	77.72	77.50	**87.50**
WDBC	97.00	96.13	**97.37**	96.49
Colon	76.92	84.62	84.62	**93.85**
Heart-Cle	57.38	58.42	70.49	**73.77**
Average	76.54	79.22	82.50	**87.90**

## Data Availability

Four tumor microarray data sets used to support the findings of this study have been deposited in the public data sources, which include UCI Machine Learning Repository and http://datam.i2r.a-star.edu.sg/datasets/krbd/.
